# III. Cellular ultrastructures *in situ* as key to understanding tumor energy metabolism: biological significance of the Warburg effect

**DOI:** 10.12688/f1000research.2-10.v1

**Published:** 2013-01-10

**Authors:** Halina Witkiewicz, Phil Oh, Jan E Schnitzer

**Affiliations:** 1 Proteogenomics Research Institute for Systems Medicine, San Diego, California, 92121, USA

## Abstract

Despite the universality of metabolic pathways, malignant cells were found to have their metabolism reprogrammed to generate energy by glycolysis even under normal oxygen concentrations (the Warburg effect). Therefore, the pathway energetically 18 times less efficient than oxidative phosphorylation was implicated to match increased energy requirements of growing tumors. The paradox was explained by an abnormally high rate of glucose uptake, assuming unlimited availability of substrates for tumor growth *in vivo*. However, ultrastructural analysis of tumor vasculature morphogenesis showed that the growing tissue regions did not have continuous blood supply and intermittently depended on autophagy for survival. Erythrogenic autophagy, and resulting ATP generation by glycolysis, appeared critical to initiating vasculature formation where it was missing. This study focused on ultrastructural features that reflected metabolic switch from aerobic to anaerobic. Morphological differences between and within different types of cells were evident in tissue sections. In cells undergoing nucleo-cytoplasmic conversion into erythrosomes (erythrogenesis), gradual changes led to replacing mitochondria with peroxisomes, through an intermediate form connected to endoplasmic reticulum. Those findings related to the issue of peroxisome biogenesis and to the phenomenon of hemogenic endothelium. Mitochondria were compacted also during mitosis. *In vivo*, cells that lost and others that retained capability to use oxygen coexisted side-by-side; both types were important for vasculature morphogenesis and tissue growth. Once passable, the new vasculature segment could deliver external oxygen and nutrients. Nutritional and redox status of microenvironment had similar effect on metabolism of malignant and non-malignant cells demonstrating the necessity to maintain structure-energy equivalence in all living cells. The role of glycolysis in initiating vasculature formation, and in progression of cell cycle through mitosis, indicated that Warburg effect had a fundamental biological significance extending to non-malignant tissues. The approach used here could facilitate integration of accumulated cyber knowledge on cancer metabolism into predictive science.

***“It isn’t what we don’t know that gives us trouble; it’s what we know that ain’t so”.***

*– Will Rogers*

## Introduction

In 1976, in his concluding remarks on Otto Warburg’s controversial theory of respiratory impairment in cancer, announced 50 years earlier, Sidney Weinhouse emphasized the need for basic knowledge of the biology of the cell as a way to elucidate the unresolved issues of “regulatory malfunctions which underlie neoplasia” ^1^. Indeed, during the following years much cancer research focused on isolated tumor cells cultured *in vitro*, analyzed by flow cytometry, subjected to molecular high-throughput genomics and proteomics analyses, or studied *in vivo* by light microscopy. The outburst of interest in oncogenes dominated over metabolic studies; however, many genetic mutations affecting signaling pathways turned out to be those involved in regulation of metabolic processes. The modern approaches contributed to currently persisting understanding that alterations in cellular metabolism should be considered one of the crucial hallmarks of cancer ^2, 3^. Yet the biological significance of the Warburg effect, the aerobic glycolysis, remained elusive and the dependence of tumor growth on glycolysis instead of oxidative phosphorylation (i.e. on the pathway with net gain of two instead of 36 ATP molecules per one molecule of glucose) remained intriguing ^3, 4^. Theoretically, the increased rate of glycolysis could compensate for the energetic imbalance; however, such reasoning is not supported by tumor hypoglycemia commonly observed *in vivo* ^2^.

Here the old controversy on the respiratory impairment in cancer is addressed from the perspective of tissue biology rather than cell biology, following recent findings regarding cellular mechanisms of tumor vasculature formation ^5, 6^. The new perspective emerged from putting *in vitro* grown mammary tumor spheroids back into the tissue context and increasing the resolution of morphological analysis *in situ* to the ultrastructural level which enabled evaluation of subtle differences in the physiological status of individual cells. Before implantation, tumor spheroids were getting all the energy they needed from properly buffered culture medium in an optimized atmospheric environment and they formed no vessels. After pseudo-orthotopic implantation, the tumors induced stem cells of homologous tissue graft to form neo-vasculature for them ^5^. The ectopic implantation put tumors in a critical situation because none of the first two options were available; the spheroids had to fend for themselves ^6^. They did so in more than one way: (1) by losing part of its population through erythrosomal autophagy, (2) by establishing paracrine dialog with initially non-responsive, non-homologous, local tissue stem cells (TSCs), (3) by self-organizing, i.e. inducing some of the tumor cells to differentiate into hematopoietic stem cells (HSCs). The third option was most astounding as it meant trans-differentiation to a nonmalignant phenotype. Until new functional vessels formed, the growing regions suffered from malnourishment and hypoxia, manifested by changes in ultrastructural features of the cells. The approach used here allowed monitoring of the earliest stages of vasculature morphogenesis occurring *in vivo* and contributed a much needed qualitatively new perspective to the importance of sub-populations of cancer cells ^7, 8^. Natural interactions among cells, within and between types, could be deduced that way and complex morphogenetic processes reconstructed in retrospect, i.e. not in real time but faithfully. That type of information is critical for the integration of multiple types of data for signaling research ^9^. It could also inspire computer modeling to add quantitative aspects to the analysis.

*In situ* ultrastructural analysis is suitable to study cellular metabolism because metabolic pathways have variable structural bases. Ultrastructures are as dynamic as the processes supported by them. The most energetically efficient pathway (oxidative phosphorylation) requires the most complex structure (mitochondrion) providing the enclosed space necessary for the existence of a proton gradient because the movement of protons across the inner mitochondrial membrane is the primary energy-conserving event. The less efficient pathway (glycolysis) occurs in cytoplasm and can be reproduced *in vitro*. Without specific protein markers, no particular glycolysis-associated structure is visualized by transmission electron microscopy (TEM) although in erythrosomes, known for using glycolysis to generate ATP ^10^, abundance of calmyrites suggested that those complexes might be involved ^5^. When not needed, mitochondria become degraded. Therefore, there is a correlation between active oxidative phosphorylation with presence of mitochondria. Uncoupling of oxidation from phosphorylation correlates with subtle but noticeable morphological changes of mitochondria as seen in brown fat ^11^.

The concept of compartmentalization of specific metabolic processes is widely accepted. A myriad of different metabolic pathways and processes associates with peroxisomes, i.e. relatively simple cytoplasmic organelles (0.1–1.0 µm in diameter), bound with a single membrane and missing DNA. The enzymatic content of such small organelles serves as a criterion to distinguish them from structurally similar, acid hydrolyses containing, lysosomes ^12^. Despite their structural simplicity, peroxisomes are surrounded by a certain aura of mystery ^13^ because of their resemblance to mitochondria with regard to several functional characteristics ^14^. The two kinds of organelles appear to cooperate. Notably, the initial steps of all very long chain fatty acid (VLCFA) metabolism occur in peroxisomes whereas the subsequent steps occur in mitochondria. The advantage of such cooperation is the protection of mitochondria from the damaging effect of by-products (free radicals) when VLCFA (longer than C-18) are broken down into chains shorter than C-9 to be exported to mitochondria ^15^. Historically, catalase activity served as the criterion identifying peroxisomes even if their 3D shape ^16^ was similar to the mitochondrial tubular network discovered later ^17– 19^. The interplay between the two organelles in peroxisomal disorders suggests more than just an overlap of functions ^14, 20^. Interestingly, they also share fission machinery; although, in addition to fission of existing organelles, peroxisomes are said to be formed *de novo* from the endoplasmic reticulum (ER) ^21– 23^. Yet, no direct evidence of physical contact *in vivo* between mitochondria and morphogenesis of peroxisomes is available, except for genetically modified cells cultured *in vitro* ^24^. The nature of those parallels between the two organelles remains unclear.

This report shows how *in situ* ultrastructural analysis proved useful for further substantiating earlier hypothesized structural relationship between peroxisomes, ER, and mitochondria ^14, 23^ and for revealing the biological significance of the aerobic glycolysis in metazoan vasculature morphogenesis and tissue growth, i.e. of the Warburg effect.

## Materials and methods

The study was performed according to protocols approved by the Sidney Kimmel Cancer Center’s (SKCC) OLAW-approved Institutional Animal Care and Use Committee (Assurance No A4128-01). The protocol numbers were: 03-16A and 05-11 for Grants CA104898 and CA119378, respectively. No human specimens were involved in any of the experiments outlined here.

A total of five recipient mice were used in the study described here and in the two accompanying articles. The same numbering system was used in all three articles. The experimental design is summarized in Table 1.

**Table 1.** 

Figure numbers	Host mouse no	Tumor cell line	Graft tissue	Incubation time (days)
8 [D]	1	N202.1A+H2B-GFP	None	21
–	2	N202.1A+H2B-GFP	Breast fat pad	21
1, 4 [C,D], 7 [A]	3	N202.1A+H2B-GFP	Breast fat pad	22
2, 3, 4 [A,B], 7 [B,C], 8 [A–C]	4	N202.1A+H2B-GFP	None	22
5, 6 [A–H]	5	N202.1A parental	Ubiquitin-GFP breast fat pad	5

### Animals

Host and graft donor female, athymic, nude mice, 8–9 weeks old, were purchased from Harlan. The donor mouse with GFP-labeled ubiquitin was from The Jackson Laboratory (Stock Number: 004353; Strain Name: C _57_BL/6-Tg(UBC-GFP) _30_Scha/J). The mice were housed in the SKCC animal care facility. For surgery, they were anesthetized (7.3 mg ketamine hydrochloride and 2.3 mg xylazine/100 g body weight, inoculated i.p.) and placed on a heating pad. Immediately before tissue harvesting the tumor hosting mice as well as the graft donors were euthanized with pentobarbital overdose (100 mg/kg i.p.).

### Cell lines

The parental murine breast cancer cell line, N202.1A ^25^ was stably transfected to express GFP under histone H2B promoter ^26^. The two cell lines, N202.1A parental and N202.1A+H2B-GFP (obtained from Drs. J. Lustgarten and P. Borgstrom) were used to form tumor spheroids by culturing 2×10 ^5^ cells per well for 2–3 days prior to implantation.

### Chambers

A week after establishment of mouse dorsal skin chambers, the tumor spheroids were implanted on a pad of homologous tissue, namely, minced breast fat pad from a lactating mouse (pseudo-orthotopically) or without graft (ectopically) as described earlier ^27^. The tumors were incubated in the chambers for one or three weeks. Their final size was about 1–3 mm in diameter.

### Antibodies

The GFP-specific rabbit polyclonal IgG (ab290) was from Abcam; CIB1-specific rabbit polyclonal IgG (11823-1-AP) was from ProteinTech Group, Inc.; CD34-specific rat monoclonal IgG _2a_ (sc-18917) and non-reactive goat polyclonal IgG (sc-34284) were from Santa Cruz.

### Tissue processing

The tumors with some surrounding tissues were dissected out and cut in halves perpendicular to the host skin surface while immersed in cold fixative (4% paraformaldehyde in 0.1 M Na cacodylate pH 7.4). The skin region served later as a reference to distinguish between the edges of the tumor facing the skin and those facing the glass window of the chamber. The halves were then separated and processed independently for TEM and immunocytochemistry.

### TEM

The tissues were transferred into a stronger fixative (4% paraformaldehyde/2.0% glutaraldehyde in 0.1 M Na cacodylate pH 7.4) to better preserve the ultrastructures before further cutting. They were cut into 1 mm thick slices in planes perpendicular to the plane of the first cut and to the skin surface, finally, into ~ 1 mm ^3^ blocks, transferred into a fresh portion of the fixative in which they were cut and incubated for 2 hrs at 4°C. The fixed tissue blocks were washed with 0.1 M Na cacodylate – HCl buffer pH 7.4 (3 × 15 min.) and post fixed in 1% OsO _4_ in 0.1 M Na cacodylate buffer, pH 7.0 for 60 min. on ice, washed with water and stained with 1% uranyl acetate at RT for one hour. The blocks were embedded in EMbed-12 (EM Sciences, Cat. No 14120). The resin embedded tissues were cut into 60 nm sections, on a Leica Ultracut UCT ultramicrotome and stained with lead citrate ^28^ or viewed without further contrasting.

### Immunocytochemistry

During cutting into ~ 1 mm ^3^ blocks as described above, the tissues were kept in the mild fixative to protect antigenic epitopes (4% paraformaldehyde in 0.1 M Na cacodylate pH 7.4). The tissue blocks were vitrified by infiltrating the pieces with 50% PVP (polyvinylpyrrolidone) containing 2.3 M sucrose in 0.1 M Na-cacodylate buffer, pH 7.4, for 2 hrs or overnight, mounted on metal pins and frozen in liquid nitrogen, as described by Tokuyasu ^29^. Frozen tissues were cut into 70 nm sections, on a Leica Ultracut UCT ultramicrotome with the cryo-attachment. The sections were picked from the knife with 2.3 M sucrose and floated on 1% ovalbumin (Sigma, Cat No.A5378) in 0.1 M Na-cacodylate buffer for at least one hour before incubation with specific or non-reactive antibody (50 µg/ml), at RT for one hour. Sections were then rinsed eight times with 0.1% ovalbumin in the same buffer and incubated for one hour with 10 nm Au coupled to protein A (from Dr G. Posthuma; Cell Microscopy Center, University Medical Center Utrecht, The Netherlands). The eight rinsing steps were repeated before fixation of the immune complexes with 1% glutaraldehyde. After rinsing three times with water, the immunostained cryosections were contrasted with a mixture of uranyl acetate and methyl cellulose (25 centipoises, Sigma M-6385) in water, at a final concentration of 1.3% each, for 10 min at RT. Excess liquid was removed and the sections were dried at RT.

### Viewing

All sections were viewed and the images captured at 100 kV using a Morgagni 268 electron microscope equipped with a MegaView III digital camera. Images were transmitted from the microscope camera to an iTEM imaging platform from Olympus Soft Imaging Solutions and archived in a designated database. In some cases, the final images were assembled by multiple image alignment (MIA) to increase the surface area without losing the resolution. We used the graphics editing program, Adobe PhotoShop, to add cell type-specific color-coding shown in the twin sets of images included in the Supplement.

## Results

**Figure 1.**
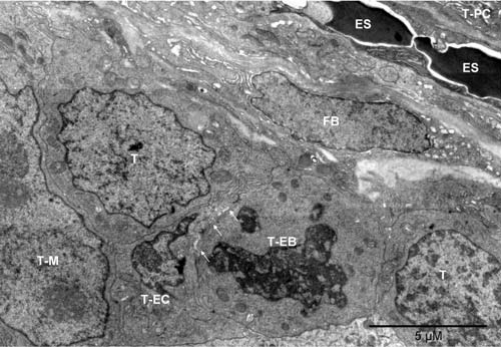
Tumor-derived vasculature precursor cells and hemogenic fibroblast. Mouse 3. The erythrosomes (ES) and the fibroblast (FB) oriented “diagonally” separated the tumor nodules. One nodule contained plasma cells (T-PC), as partly shown in the upper right corner. Cells in the lower and left part of the image belonged to the other nodule. Flanked by tumor cells were the vasculature-initiating cells: erythroblast (T-EB), as identified by the disappearing nucleus and mitochondria converting into peroxisomes, and endothelial cell (T-EC). The two cells were locally separated by anoikis (arrows) ^30^. To the left of the T-EC, is a partly shown large nucleus with two nucleoli, likely belonging to a megakaryocyte of tumor origin (T-M); peroxisomes are visible in the cytoplasm above the nucleus.

**Figure 2.**
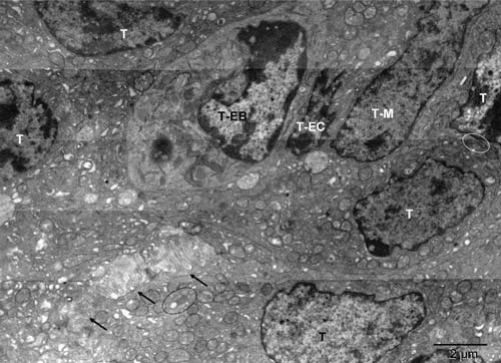
Tumor-derived vasculature precursor cells (II); microenvironment of the cell shown in Figure 3. Mouse 4. The erythroblast (T-EB), differentiating endothelial cell (T-EC), possibly megakaryocyte (T-M). Ovals contain peroxisomes forming in tumor cells (T). Arrows point to the anoikis ^30^ area.

**Figure 3.**
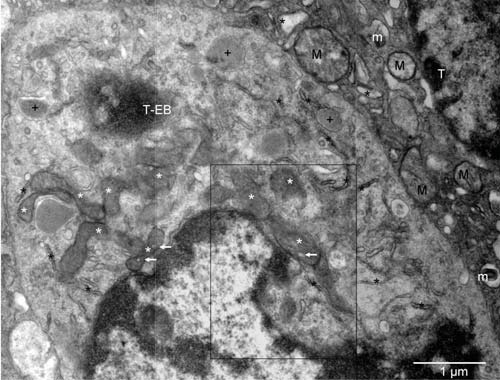
Biogenesis of peroxisomes in tumor-derived erythroblast. Mouse 4. Mitochondria at various stages of conversion into peroxisomes (white stars); ER (black stars); autophagosomes (+). The arrows point to electron lucent vesicles that might contain catalase (compare with Figure 7 in ^16^). The enlarged boxed area is rotated left in Figure 4[A] whereas the microenvironment of the erythroblast (T-EB) is shown in Figure 2. Mitochondria (M) of the tumor cell (T) in the upper right corner are characteristic of the quiescent cells but some show signs of hypoxia (m), as do dilated cisternae of ER (black stars).

**Figure S1.**
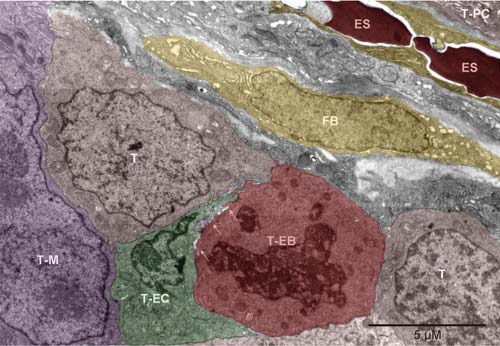
Tumor-derived vasculature precursor cells and hemogenic fibroblast. Mouse 3. The erythrosomes (ES, red) and the fibroblast (FB, yellow) oriented “diagonally” separated the tumor nodules. One nodule contained plasma cells (T-PC, brown), as partly shown in the upper right corner. Cells in the lower and left part of the image belonged to the other nodule. Flanked by tumor cells (T, brown) were the vasculature-initiating cells: erythroblasts (T-EB, red), as identified by the disappearing nucleus and mitochondria converting into peroxisomes, and endothelial cell (T-EC, green). The two cells were locally separated by anoikis (arrows) ^30^. To the left of the T-EC, is a partly shown large nucleus with two nucleoli, likely belonging to a megakaryocyte of tumor origin (T-M, purple); peroxisomes are visible in the cytoplasm above the nucleus.

Formation of vasculature in the avascular tumor spheroids has been shown to depend in our model on the presence of the homologous tissue graft which results in faster tumor growth ^5, 6^. However locally, the phenomena described for the ectopically implanted tumors (Figure 1–3 in ^6^) also occurred in the presence of the graft, i.e. after pseudo-orthotopic implantation (see Table 1 to correlate specific images with particular mice). Those phenomena were the early stages of vasculature formation within tumor nodules and the erythrogenesis in fibroblasts separating the nodules, which we have demonstrated to highlight the variable dynamics of the tumor microenvironment *in situ*. A typical hematopoietic cell triplet, megakaryocyte, erythroblast and EC (with anoikis between the last two cells ^30^) located inside the tumor nodule, suggests that some tumor cells were evolving into re-differentiated (non-malignant) cells ( Figure 1 and Figure S1). The erythroblast with depleted mitochondria and accompanied by an EC and a megakaryocyte coexisted side by side with tumor cells displaying the pattern of mitochondrial profiles typical of the resting phase of the cell cycle (fragmented mitochondria ^31^). Such ultrastructural variability would not be possible if all cells used the same type of metabolism, like yeast in suspension do, depending only on the environmental conditions. Therefore, that image provided an answer to the quest for tumor-specific defects of mitochondria. The loss of mitochondria must have resulted in impairment of respiration in erythroblasts and platelets but not in the cells that maintained them. The phenomenon was not tumor-specific. The fibroblasts squeezed between the tumor nodules were also suffering from local hypoxia, and some of them were also converting into erythrosomes (in a slightly different way).

**Figure S2.**
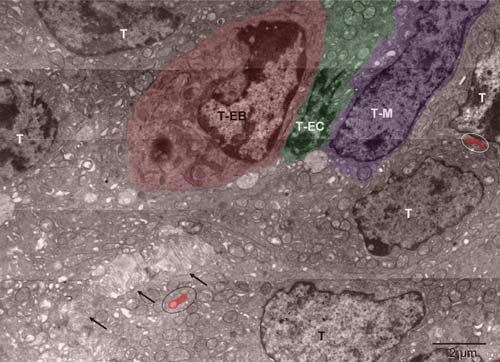
Tumor-derived vasculature precursor cells (II); microenvironment of the cell shown in Figure 3. Mouse 4. The erythroblast (T-EB, red), differentiating endothelial cell (T-EC, green), possibly megakaryocyte (T-M, purple). Ovals contain peroxisomes (red) forming in tumor cells (T, brown). Arrows point to the anoikis ^30^ area.

**Figure S3.**
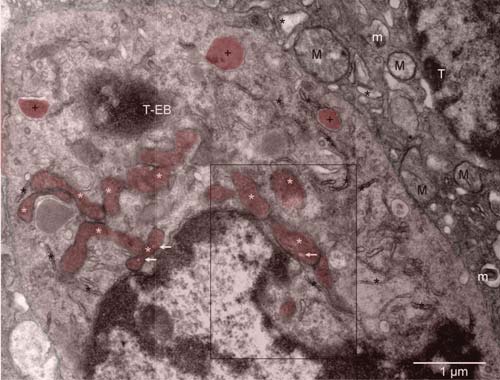
Biogenesis of peroxisomes in tumor-derived erythroblast. Mouse 4. Mitochondria at various stages of conversion into peroxisomes (white stars); ER (black stars); autophagosomes (+). The arrows point to electron lucent vesicles that might contain catalase (compare with Figure 7 in ^16^). The enlarged boxed area is rotated left in Figure S4[A] whereas the microenvironment of the erythroblast (T-EB, reddish brown) is shown in Figure S2. Mitochondria (M) of the tumor cell (T) in the upper right corner are characteristic of the quiescent cells but some show signs of hypoxia (m), as do dilated cisternae of ER (black stars).

The erythroblast located in another tumor nodule represented an earlier stage of the erythrogenic conversion ( Figure 2 and Figure S2). The nucleus of that cell was more prominent despite having integrity compromised by the local absence of a nuclear membrane and invasion of the cytoplasm. Mitochondria were morphologically different from the fragmented ones in the surrounding tumor cells and highly pleomorphic ( Figure 3 and Figure S3). Their profiles resembled those of the organelles in partly differentiated cells (type II) of the mouse preputial gland, where they contained catalase and were thought of as peroxisomes (Figure 1 in ^16^). Both cell types featured mitochondria undergoing the process of conversion into peroxisomes. The mitochondrial properties were: double outer membrane and internal cristae. The peroxisomal-like properties were degradation of the cristae, increasing electron density, remodeling of the double outer membrane into the single membrane, and internal vesicles like those containing catalase. By extrapolation, the 2D pleomorphic profiles in the erythroblast (meta-chondria and peroxi-chondria) shared the 3D structure with the “peroxisomes” of the type II preputial gland cell ^16, 17^ and with the mitochondria ^17, 18, 31^.

**Figure 4.**
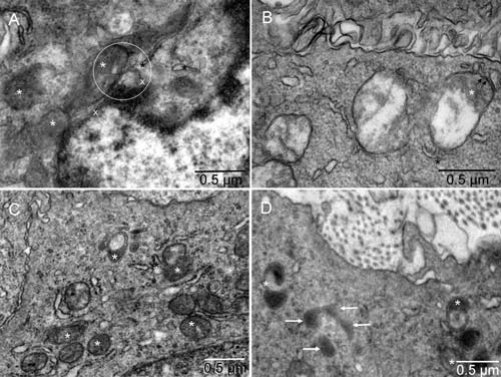
Biogenesis of peroxisomes. Mouse 4. The point of contact for mitochondrial outer membrane (white star), ER (black star) and nuclear membrane (x) is located in the center of the circle [ **A**]. Such membrane continuity between the ER and mitochondrion converting into peroxisome was also seen in a tumor cell with intact nucleus [ **B**]. Examples of more advanced forming peroxisomes are shown in [ **C** & **D**]. Arrows point to peroxisomes with a single membrane [ **D**].

**Figure 5.**
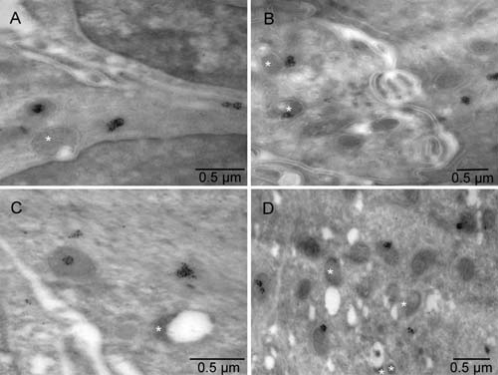
Calmyrites in non-dividing tumor cells with variable morphology of mitochondria. Mouse 5. Electron lucent regions were commonly associated with atypically shaped mitochondria (stars). Calmyrites were detected by immunocytochemical staining with calmyrin-specific antibody.

**Figure 6.**
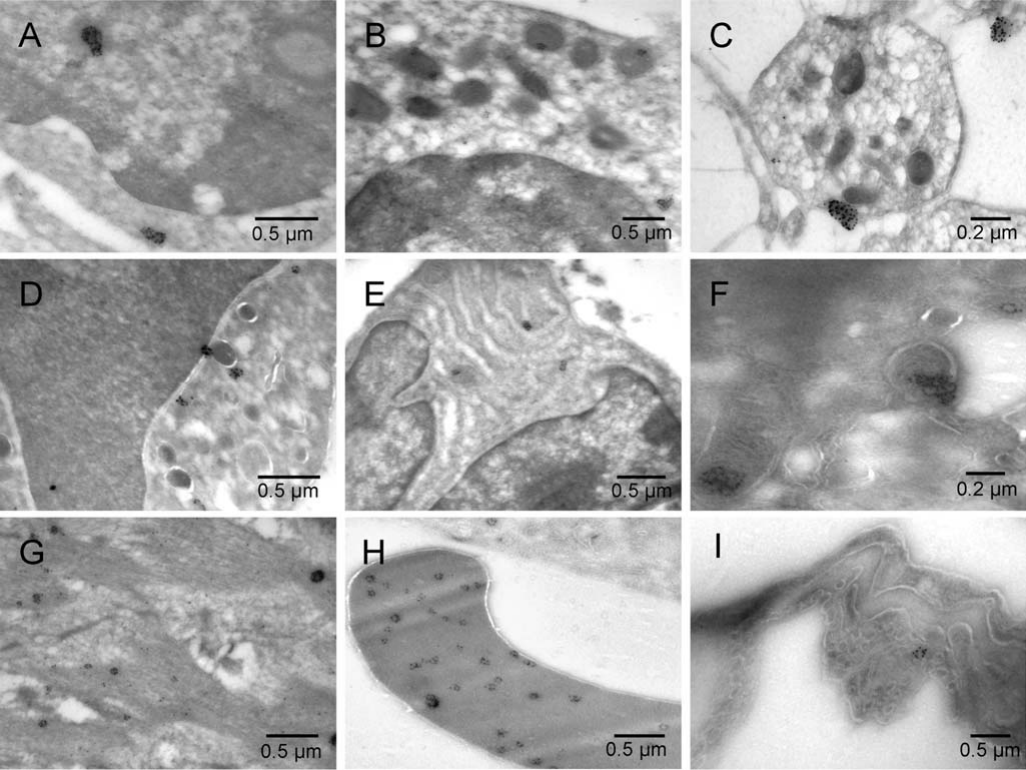
Calmyrites in different cell types. Mouse 5 [ **A**– **H**] and normal rat lung [ **I**]. Erythroblast [ **A**], megakaryocyte [ **B**], platelet [ **C**], granulocyte [ **D**], plasma cell [ **E**], hemangioblast [ **F**], smooth muscle [ **G**], erythrosome [ **H**], and lung endothelium of control sample from normal rat lung [ **I**].

**Figure 7.**
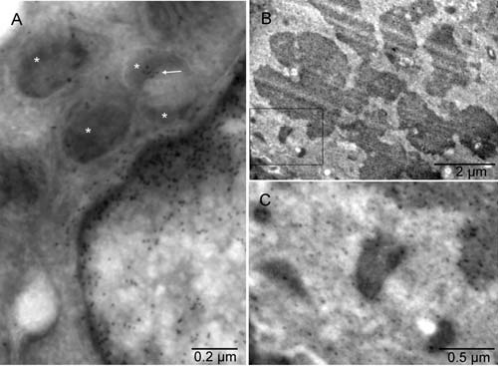
H2B-GFP label in tumor cells at various phases of the cell cycle. Mouse 4. Gold grains associated with chromatin of the interphase nucleus and a cluster of the gold particles (arrow) co-localized with one of the peroxisomes (stars) in [ **A**]. The label was also found in the mitotic chromosomes and mitochondria of the dividing cell [ **B**]. In the cell undergoing mitosis, the mitochondria were comparatively smaller, more electron-dense and often accompanied by an electron-lucent region, similar to those converting into peroxisomes [ **C**]. Mice 3 [ **A**] & 4 [ **B**& **C**].

**Figure 8.**
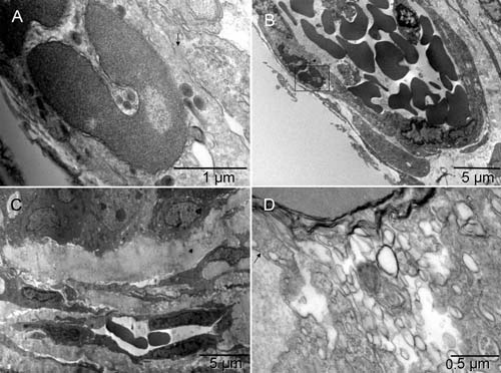
Hemogenic endothelium. Mouse 4. The cell partly shown in [ **A**] (enlargement of the boxed area in [ **B**]) displayed features typical of definitive erythroblasts undergoing nucleo-cytoplasmic conversion: dark nuclear lobes separated from cytoplasm by multilayered corrugated membranes and peroxisomes in the absence of mitochondria. However, the shape and location of that cell were such that by light microscopy, it would look like an EC [ **B**]. At one region, its plasma membrane appeared fused with the membrane of an underlying EC (arrow in [ **A**]) suggesting that simultaneously with experiencing erythrogenic conversion, the cell was penetrating the endothelium of a vessel that already contained some non-circulating blood (as judged by irregular shapes of the erythrosomes). A smaller, forming vessel in [ **C**] appeared to have converted one of its’ ECs into erythroschizosome, which had then moved into the lumen, leaving a vacant space behind, and had already separated one monomer completely and another one partially. The cell in [ **D**] represented an ambivalent (hemangioblastic) phenotype, similar to those seen five days after pseudo-orthotopic implantation ^5^. However, here it had an increased level of phenotypic complexity, as manifested by the presence of caveolae (arrow), a hallmark of EC, and remodeling of the internal membranes covering the emerging erythrosome (upper left corner), in addition to collagen synthesis (bundle of collagen fibers under the right end of the scale bar).

**Figure S4.**
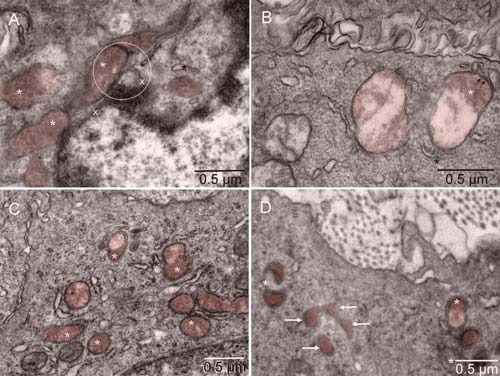
Biogenesis of peroxisomes. Mouse 4. The point of contact for mitochondrial outer membrane (white star), ER (black star) and nuclear membrane (x) is located in the center of the circle [ **A**]. Such membrane continuity between the ER and mitochondrion converting into peroxisome was also seen in a tumor cell with intact nucleus [ **B**]. Examples of more advanced forming peroxisomes are shown in [ **C** & **D**]. Arrows point to peroxisomes with a single membrane [ **D**]. Mitochondria and peroxisomes are in different shades of brown in comparison to the rest of each cell.

**Figure S5.**
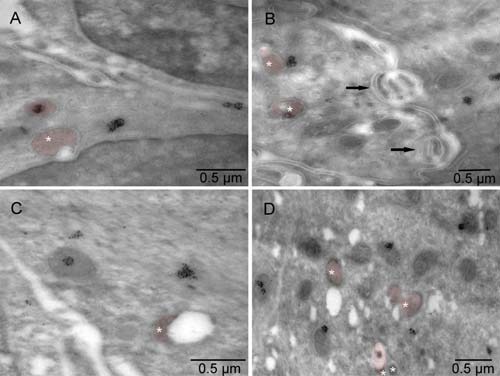
Calmyrites in non-dividing tumor cells with variable morphology of mitochondria. Mouse 5. Electron lucent regions were commonly associated with atypically shaped mitochondria (brown). Calmyrites were detected by immunocytochemical staining with calmyrin-specific antibody. Arrows indicate anoikis.

**Figure S6.**
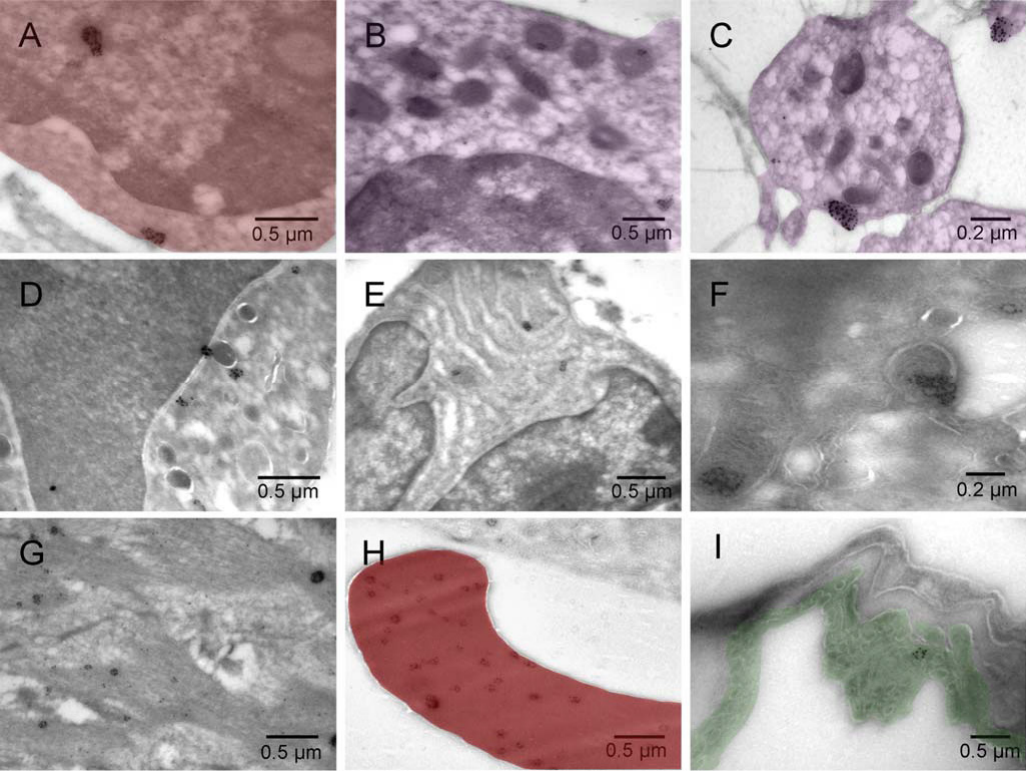
Calmyrites in different cell types. Mouse 5 [ **A**– **H**] and normal rat lung [ **I**]. Erythroblast [ **A**, red], megakaryocyte (purple) [ **B**], platelet (purple) [ **C**], granulocyte [ **D**], plasma cell [ **E**], hemangioblast [ **F**], smooth muscle [ **G**], erythrosome (red) [ **H**], lung endothelium (green) of control sample from normal rat lung [ **I**].

**Figure S7.**
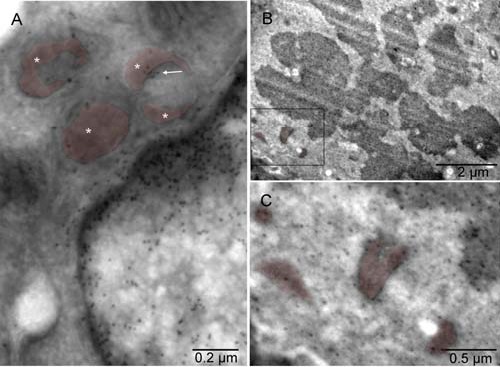
H2B-GFP label in tumor cells at various phases of the cell cycle. Mouse 4. Gold grains associated with chromatin of the interphase nucleus and a cluster of the gold particles (arrow) co-localized with one of the peroxisomes (stars) in [ **A**]. The label was also found in the mitotic chromosomes and mitochondria of the dividing cell [ **B**]. In the cell undergoing mitosis, the mitochondria were comparatively smaller, more electron-dense and often accompanied by an electron-lucent region, similar to those converting into peroxisomes [ **C**]. Mice 3 [ **A**] & 4 [ **B & C**]. Mitochondria and peroxisomes are in different shades of brown.

The early erythroblast contained a region displaying continuity of the membranes from three kinds of organelles. Those organelles were nucleus, mitochondrion and ER ( Figure 4[A] and Figure S4[A]). In the tumor cell with intact nucleus (not committed to erythrogenesis), the nuclear membrane was not involved but the membrane continuity between the ER and the deteriorating mitochondrion was seen ( Figure 4[B] and Figure S4[B]). The continuity of the membranes indicated their gradual remodeling rather than complete degradation of the existing system of membranes before synthesizing the new, evolving type(s) ^5^. The irregular mitochondrial shape, gradual disassembly of mitochondrial cristae and condensation of the remaining components into an electron-dense region enclosed by a single membrane indicates a morphogenetic relationship with peroxisomes ^12^. Examples of more advanced stages of peroxisomal morphogenesis are shown in Figure 4[C & D] and Figure S4[C & D]. Calmyrites, the para-crystalline structures detectable with calmyrin-specific antibodies and abundant in erythrosomes ^5^, were occasionally associated with mitochondria and with the intermediate forms ( Figure 5 and Figure S5). In addition to tumor cells, they were seen in erythroblasts, megakaryocytes, platelets, granulocytes, smooth muscle cells, plasma cells ( Figure 6 and Figure S6) and in the epithelium and endothelium of the control sample from normal rat lung, (Figure 8[G] in ^5^ and Figure 6 and Figure S6 [I], respectively). In megakaryocytes and platelets, they co-localized with peroxisomes ( Figure 6[B & C] and Figure S6[B & C]). The histone H2B-specific label detected chromatin in the nucleus and some in one of the coffee bean-resembling, “twin” peroxisomes next to it ( Figure 7[A] and Figure S7[A]).

Condensation and an increase in electron density of mitochondria during mitosis suggested a lowered rate of respiration at that phase of the cell cycle (Figure 7[B & C] and Figure S7[B & C] and Figure 2[F] in ^6^). At mitosis, the mitochondria were not just fragmented as during the interphase ^19^ but compacted, electron dense, resembling peroxisomes yet capable of reversing those morphological changes. The structural difference between the mini-chondria seen at mitosis and the fragmented eu-chondria during the resting phase of the cell cycle was obvious at the resolution of TEM. It was missed by fluorescent microscopy.

**Figure S8.**
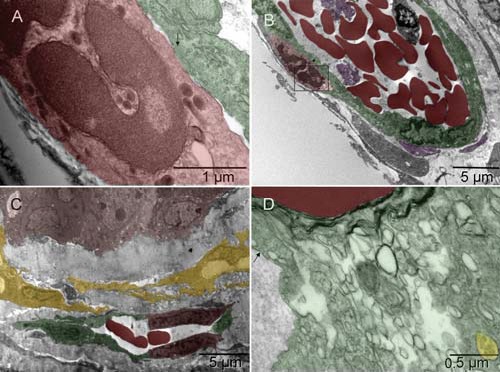
Hemogenic endothelium. Mouse 4. The light red cell partly shown in [ **A**] (enlargement of the boxed area in [ **B**]) displayed features typical of definitive erythroblasts undergoing nucleo-cytoplasmic conversion: dark nuclear lobes separated from cytoplasm by multilayered corrugated membranes and peroxisomes in the absence of mitochondria. However, the shape and location of that cell were such that by light microscopy, it would look like an EC [ **B**]. At one region, its plasma membrane appeared fused with the membrane of an underlying green EC (arrow in [ **A**]) suggesting that simultaneously with experiencing erythrogenic conversion, the cell was penetrating the endothelium of a vessel that already contained some non-circulating blood (as judged by irregular shapes of the erythrosomes). A smaller, forming vessel in [ **C**] appeared to have converted one of its EC into erythroschizosome (red), which had then moved into the lumen, leaving a vacant space behind, and had already separated one monomer completely and another one partially. Fibroblasts and tumor cells are shown in yellow and brown, respectively. The cell in [ **D**] represented an ambivalent (hemangioblastic) phenotype, similar to those seen five days after pseudo-orthotopic implantation ^5^. However, here it had an increased level of phenotypic complexity, as manifested by the presence of caveolae (arrow), a hallmark of EC (green), and remodeling of the internal membranes covering the emerging erythrosome (red in upper left corner), in addition to collagen synthesis (yellow bundle of collagen fibers under the right end of the scale bar).

If forming vessels did not fuse with the circulatory system before the local environment ran out of oxygen and nutrients, even the differentiated ECs would undergo erythrogenic autophagy ( Figure 8 and Figure S8). Within the EC type, individual cells behaved variably; the effect of hypoxia was not synchronous. That way some cells could help others to survive longer, although they perished in the process of nucleo-cytoplasmic conversion. Upon a significant change of the redox status, one could envision the surviving ECs guiding the differentiation of other cells into phenotypes matching their level of complexity ^5^. Thus, erythrogenesis could be induced by factors limiting metabolic processes due to hypoxia and malnutrition resulting from either tissue growth (developmental and malignant) or the opposite, i.e. failure of forming vessels to continue growing until they fused with the circulatory system. Hypoxia and a shortage of nutrients affected all cell types in that microenvironment; cell type-dependent differences were subtle ( Figure 1 and Figure S1). Non-malignant, collagen-producing fibroblasts outside the blood oxygen diffusion range converted most, but not all, of their bodies into erythrosomes; remnants of the cytoplasm remained, and sometimes mitochondria were present during the ongoing erythrogenic process ( Figure 3 in ^6^). To the contrary, erythroblasts of tumor origin were undergoing a complete erythrogenic conversion whilst also initiating the vasculature formation-related cascade of differentiation within the tumor population. With regard to their ability to produce erythrosomes under stressful conditions, those examples were not different from hemogenic endothelium ^32^.

## Discussion

### Experimental approach

Systems biology research struggles with a paucity of satisfactory methods because local micro-environmental differences, meaningful *in vivo*, are hard to reproduce *in vitro* where conditions controlled experimentally affect the analyzed phenomenon indiscriminately. For example, purified DNA molecules can easily be condensed *in vitro* by dehydration and charge neutralization (adding alcohol and salt, respectively) but the entire length of each molecule is affected simultaneously ^33^. However *in vivo*, the process is controlled in a more shrewd way (through protein-DNA interactions), assuring local conformational differences within a single molecule ^34^. Similarly, cells isolated from tissues and cultured *in vitro* lose their original functional characteristics. To study cellular interactions, preserving tissue structure is necessary. The *in situ* analysis of ultrathin tissue sections enabled the examination of complex cellular interactions that would have been missed by other methods. It also allowed dialectical interpretation of the observed phenomena that appeared contradictory when studied independently. That approach exposed new relationships between intra- and inter-cellular events and implied logical connections between tissue morphogenesis and metabolic pathways. *In vivo*, cells with different types of metabolism were located side-by-side and cooperated ( Figure 1, Figure 2, Figure S1 and Figure S2). Therefore, in addition to environmental factors (hypoxia and hypoglycemia) that triggered the metabolic switch from oxidative phosphorylation to glycolysis in some cells, tissue-intrinsic regulatory mechanisms must have been involved to control types of metabolism for each cell individually, yet cooperatively for all. The environmental factors triggered the response, but tissue-inherent factors acted as modulators that made the response differential and resulted in changes most beneficial for the tissue rather than individual cells. The *in situ* analysis demonstrated great heterogeneity of cellular phenotypes within relatively small tissue fragments. Characterizing metabolic pathways of those individual cell types without changing their properties would not have been possible by methods requiring destruction of the tissue fabric. Such relational characterization is necessary to unravel metabolic processes occurring *in vivo*.

### Extramedullar erythrogenesis and tumor cellular heterogeneity

The single piece of information that was most consequential for viewing tumor energy metabolism from a new perspective was the one on the mechanism of erythrogenesis ^5^. It showed how the conclusions regarding “erythrocytic enucleation” in other models ^35, 36^ had a long-lasting misleading effect on studies of tumor vasculature morphogenesis. While the presence of non-malignant cells of hematopoietic lineage in tumor samples had been known for years, it was difficult to explain. They were assumed to be “recruited to” or “extravasated at” the tumor site ^3^. However, erythroblasts were not identified in tumors until recently ^5^; even though, the morphology of extramedullar erythroblasts ( Figure 1, Figure S1, Figure 2, Figure S2, Figure 6[A] and Figure S6[A]) was no different from those in human bone marrow ( *medulla ossea*) seen earlier ^12^. Previously, the megakaryocyte, erythroblast & EC triplets, as well as fibroblasts, were categorized as stromal cells when seen in tumor nodules. Here, the gradual shrinking and disappearance of mitochondria with a simultaneous proliferation of peroxisomes was correlated with the metabolic switch. That led to a glycolytic pathway in mature erythrosomes through a phase of extensive, peroxisomes-requiring restructuring of the entire cell. That phase lasted long enough to make cells with peroxisomes in the absence of mitochondria commonly encountered. The distinct morphology of the erythroblasts going through the nucleo-cytoplasmic conversion conceptually steered the erythrogenesis process from bone marrow to any location where tissue growth was happening. Practically, it helped in identifying specific locations where vasculature morphogenesis started *in vivo* (via trans-differentiation of designated cells into HSCs and their interactions with other cells) and in following the process as it unfolded. Clearly, the absence of mitochondria in erythroblasts was a good reason for their respiration to be impaired and replaced by the alternative pathway. That is why using isolated mitochondria to search for causes of the respiratory impairment was not successful, and why metabolic studies on tissue slices were showing both pathways.

### Biogenesis of peroxisomes

The nucleo-cytoplasmic conversion that we observed into erythrosomes, being the extreme case of cellular remodeling, augmented the role of peroxisomes in erythrogenesis and helped us to demystify their biogenesis considerably. The process depended on fundamental restructuring of the entire cell in a controlled purposeful fashion. It required reduction of all organelles and membranes to their primary components from which to form erythrosomes - the new organelles with their own specialized membrane. Peroxisomes, known for homing catabolic as well anabolic processes, played a critical role in the restructuring. The results were consistent with the three hypothesized sources of lipids and proteins for the creation of peroxisomal membrane namely mitochondria, ER and cytoplasm ^23^. However, in cells committed to the erythrogenic autophagy, the nucleus also contributed to the biogenesis of peroxisomes and ultimately erythrosomes ( Figure 4[A] and Figure S4[A]).

The mitochondrial reticulum was shown to be present in cultured cells only at certain phase of the cell cycle when the energy output was the greatest, just before synthesis of DNA (G1/S) ^31^. It could also be induced by starvation and reactive oxygen species ^37, 38^. Here, we present similar mitochondrial forms *in situ*, in a tumor cell that was not preparing to replicate because its nucleus was partly degraded and an early EC and potential megakaryocyte accompanied that cell. Together, the three cells represented the typical hematopoietic triplet. The erythrosomal conversion of the nucleated cells into the organelles containing no visible internal ultrastructures (erythrosomes) involved degradation of all organelles originally present in the erythroblasts ^5^. The molecular products of such degradation became substrates for the formation of the erythrosomes. The tumor cell in Figure 3 and Figure S3 was in the transition phase and so were its organelles. Mitochondria were converting into peroxisomes, and ultimately the peroxisomes would degrade as well. Hypoxia and starvation could have induced those ultrastructural changes by triggering initiation of erythrosomal authophagy.

The profiles of mitochondria were unlike those fragmented ones in the surrounding tumor cells (eu-chondria). The term meta-chondria describes them better because they were an equivalent of the peroxi-chondria in the secretory cells of glands like the preputial ^16^, Meibomian ^39^ or those producing gastrointestinal secretions ^40^. One can observe that the filamentous mitochondria generating more ATP than any other form ^31^ appear when entire cell content requires restructuring, either due to proliferation or conversion of the cell into something different, even if only gradually; for example, the erythrosomes or fat-containing vacuoles. A major similarity between the two cell types harboring such events is that both eventually perish in the process of producing sub-cellular elements: erythrosomes or lipid droplets, respectively. The partly differentiated cells of the mouse preputial gland (type II) ^16^ were in the process of differentiating into lipid-producing type III and subsequently would degenerate via the lethal cells (type IV) ^39^.

To form peroxisomes, certain elements of mitochondria were utilized, for example those supporting the overlapping functions, including fission of the organelle ^22^, but others had to be synthesized; hence, the involvement of rough ER. The conversion to peroxisomes appeared simultaneous with division of the mitochondrion. The division started by mitochondrion could be completed by peroxisomes. The continuity of partially disassembled mitochondrial membranes with ER ( Figure 4[A & B] and Figure S4[A & B]) and localization of histone H2B in the peroxisome ( Figure 7[A] and Figure S7[A]) corroborated the close relationship between mitochondria and peroxisomes ^41^ and provided evidence for direct structural connection *in situ*. Chromatin was shown here for the first time in peroxisomes, confirming their derivation from mitochondria. A very small amount of mitochondrial DNA found earlier in the preparation of isolated peroxisomes was dismissed as contamination ^17, 42^. Participation of the cytoplasmic components could only be assumed here, except for calmyrites that were usually free in cytoplasm, but in some cells commonly co-localized with peroxisomes and mitochondria ( Figure 5, Figure S5, Figure 6 and Figure S6). Substrates catabolized in peroxisomes should be those other than glucose (including amino acids, fatty acids and lactate ^43– 45^), because glycolysis does not require structural isolation from cytoplasm except in some protozoa where specialized peroxisomes are equipped with glycolytic enzymes ^14^. Therefore, the significance of co-localization of calmyrites with peroxisomes in platelets remains to be clarified.

Mitochondrial conversion into peroxisomes, correlating with hypoxia and cellular differentiation into erythroblasts, reflects concomitant metabolic changes. Erythroblasts and neurons appear to be two extreme examples with regard to their type of metabolism and peroxisomal functions. In the former, peroxisomes are more abundant than mitochondria and eventually replace them, and in the latter, using mainly glucose as a substrate for OXPHOS, peroxisomes are absent ^15^. In white blood cells, peroxisomes are common and unaccompanied by mitochondria (similar to erythroblasts), indicating that fluctuating oxygen pressure in circulating blood is incompatible with OXPHOS. Some ill-defined granules of white blood cells may represent variants of peroxisomes; a hint for such a possibility was provided by mitochondrial crystalline inter-membrane inclusions in gliomas ^46^.

### Hemogenic endothelium

According to the concept of “hemogenic endothelium”, blood derives from endothelium ^32^ because the hemogenic embryonal tissue is the tissue from which the aorta develops. The term implies that the endothelium exists before the emergence of blood cells. However, the embryonal “hemogenic endothelium” is not the kind of endothelium present in mature vascular system. As portrayed in gerbil embryo, it morphologically resembled tumor cells rather than the specialized lining of the luminal surface of blood vessels ^47^. The essential ultrastructural feature of the embryo shared with the tumor is side-by-side location of cells with differentially modified mitochondria. By contrast, the hallmarks of ECs are a flattened shape, polarized cell membrane (luminal and abluminal), abundant caveolae (mostly on luminal surface), attachment to collagen or basement membrane (on abluminal surface), collagen synthesis and Weibel-Palade bodies. Those bodies have a functional characteristic overlapping with that of alpha granules in platelets, namely involvement in production, storage and secretion of von Willebrand factor which is essential for blood coagulation ^48^.

Alternatively, ECs could evolve from hemangioblasts and possibly megakaryocytes and collagen producers (at a higher level of complexity). As with erythrogenesis, differentiation of ECs appears to occur in multiple ways ^5^. In our studies, tumor spheroids presented themselves as an analog of undifferentiated embryonic tissue but with a dysfunctional morphogenetic control system, except for vasculature that does make tumors vulnerable to natural selection. Erythrogenesis was a primary event; endothelium evolved next, contrary to the hemogenic endothelium concept, if that concept is taken literally. When comparing the tissues rather than words, one would object not to the “hemogenic” part of the term, but to the word “endothelium”. Yet, under hypoxia, the endothelium could become hemogenic ( Figure 8 and Figure S8) just like fibroblasts and tumor cells ( Figure 1, Figure S1, Figure 2 and Figure S2). By converting differentiated ECs into erythrosomes, the vessel formation could be disturbed before completion, illustrating how the environment could dominate the intrinsic potential to grow and develop ( Figure 8 and Figure S8).

### Mini-mitochondria and glycolysis at the mitotic phase of the cell cycle

Glycolysis was detected in mammalian embryos ^49, 50^ when the size of the embryo exceeded the oxygen diffusion range and blood islet appeared (Figure 6(B) in ^51^), marking the initiation of vasculature development. Activation of glycolysis in non-embryonal proliferating cells has also been known for years, for example in murine splenic lymphocytes *in vivo* ^52^, in cultured rat thymocytes ^53– 55^ or mouse lymphocytes ^56^, and in rapidly proliferating prostate cells ^57^). The energy required for nuclear division was reported to be wholly or partly derived from the anaerobic metabolism of glucose ^58^. The reduced size and the increased electron density of mitochondria at mitosis (mini-chondria) suggests that ATP generation by OXPHOS was down-regulated intermittently, with a frequency determined by the length of the cell cycle ( Figure 7[B & C] and Figure S7[B & C]). For that reason, inhibiting glycolysis with the intent to stop vasculature formation could have side effects comparable to chemotherapies targeting cell proliferation.

### Biological significance of the Warburg phenomenon

Otto Warburg deserves credit for discovering mammalian glycolysis in 1923 despite it being a minor pathway at tissue level, even if in tissue slices it was enhanced experimentally by cutting off the circulation and providing excess glucose. Warburg was aware of cellular heterogeneity in the tissue slices and thought that solid tumors had mixed metabolic pathways active because of their impurities, i.e. containing non-tumor cells. Concerned with that “contamination”, after 1950 he studied only ascites tumor cells, believing they represented a homogenous population of cells with impaired respiration ^59^. Sidney Weinhouse also used tissue slices and ascites tumor cells to study the same metabolic processes but his technique, employing isotopicly labeled metabolites, was much better suited for quantitative analysis and more sensitive in detecting the two pathways, glycolysis and oxidative phosphorylation, in comparison to the Warburg’s manometric method. He debated Warburg’s interpretation repeatedly ^1, 60– 62^. The glycolytic process was likely up-regulated in those cells in cyclical, non-synchronous fashion, due to proliferation ^63^ and variable level of hypoxia, depending on the volume of the ascites *in vivo*, as reflected by morphology of the mitochondria later visualized in ascites tumor cells by TEM ^64, 65^. Here, *in situ* ultrastructural analysis showed the metabolic differences among neighboring cells, also of the same type. Moreover, it suggested co-existence of the two metabolic pathways even within single cells, based on co-existence of mitochondria and calmyrites either co-localizing with mitochondria or free in the cytoplasm ( Figure 5, Figure S5, Figure 6 and Figure S6). The central role of erythrogenesis in vasculature formation deepened our understanding of the relationship between tissue growth and the energy aspect of metabolism by delivering the critical missing element necessary to make the connection between processes occurring at molecular and tissue levels: glycolysis and tissue morphogenesis, including malignancy. Tumors appear to be autistic with regard to interacting with morphogenetic control signals from the system of which they are a part, but capable of basic morphogenetic functions related to vasculature formation; hence their cellular heterogeneity. Otherwise, they would not grow.

The problem with cancer cells is that in many respects they are not different from non-malignant cells. Glycolysis, the less energetically efficient metabolic pathway, appeared critical as a physiological temporary alternative when oxidative phosphorylation diminished due to either growth-related hypoxia or cytokinesis-related slowdown of anabolic processes at the mitotic phase of the cell cycle. It was not tumor specific. Not even yeast could survive for long time on glycolysis alone ^59^. The two metabolic pathways were not mutually exclusive. If the biological significance of aerobic glycolysis *in vivo* was to address the needs of growing tissues ^4^, the goal was accomplished indirectly, by inducing vasculature formation and by compensating for cyclically down-regulating mitochondrial activity during mitosis. Regardless of the role that the Warburg effect might play in carcinogenesis ^59^, the phenomenon discovered by him appeared to be of profound significance for tissue morphogenesis in general, not only in malignancy. By proposing a fundamental role of metazoan glycolysis in establishing the circulatory system critical for fueling the metabolism of complex organisms, that conclusion indeed brings Warburg’s “anerobic glycolysis” to a renewed prominence, as anticipated by Weinhouse. The essential issues of “regulatory malfunctions which underlie neoplasia” remain unresolved but the level of our thinking about them has changed ^1^.
