# Supplementary material for: III. Cellular ultrastructures in situ as key to understanding tumor energy metabolism: biological significance of the Warburg effect
Source: F1000Res. 2013 Jan 10;2:10. [Version 1] doi: 10.12688/f1000research.2-10.v1 (PMC3829121; doi:10.12688/f1000research.2-10.v1)
Supplement: Peer review history

# Peer review: Referee response for version 1

**Published:** 2013-05-30

**Authors:** Karolina Kucharova

**Affiliations:** 1 Sanford-Burnham Medical Research Institute, La Jolla, CA, USA

The title: “III. Cellular ultrastructures *in situ* as key to understanding tumor energy metabolism: biological significance of the Warburg effect” is the appropriate for the content of the article.  

The abstract seems longer than needed – I suggest focusing on the observed findings of the present study.

The methods are well documented, except there is little information about the chamber and how cells were implanted into the chamber. 

In the results section, I suggest:

- Showing the mitochondrial profiles typical of the resting phase of the cell cycle (fragmented mitochondria) described in the results section
- Adding an enlarged figure of mitochondria which is converting into peroxisomes in the erythroblasts described in the legend of the figure 1., peroxisomes forming in the tumor cells described or showed in the figures 1, 2.
- Showing the mini-chondria and eu-chondria described in the results section  

I have read this submission. I believe that I have an appropriate level of expertise to confirm that it is of an acceptable scientific standard.

# Peer review: Referee response for version 1

**Published:** 2013-04-29

**Authors:** Janusz Byczkowski

**Affiliations:** 1 Ohio Environmental Protection Agency, Division of Environmental Response and Revitalization, Columbus, OH, USA

This manuscript is part three in a series of three articles, which taken together postulate a novel and intriguing hypothetical mechanism of 'vasculature morphogenesis' in tumors involving formation of blood and vessels from local tissue stem cells. The reviewed manuscript, as written, is in some places difficult to read, as the authors tend to discuss several issues at once and often jump from subject to subject, generating sometimes a little bit of narrative chaos. Also, this manuscript may be difficult to comprehend without the context of the remaining two companion articles and quoted references. For this reason, I would suggest, that all three articles should have been published together. Moreover, another problem with this kind of functional morphology research is that the researchers have to present their observational study describing what they see, interpreting pictures qualitatively and deriving working hypotheses without translating visual fields into numbers and supporting statistics, which would be meaningless in this study, as there are no conflicting hypotheses to test, or control group to compare. 

Anyway, in this manuscript, the authors could more clearly separate their own results from extended discussions (especially in the “Results” section), and perhaps, could easily cut from their interpretations some embellishments, like for example “... *The approach used here could facilitate integration of accumulated cyber knowledge on cancer metabolism into predictive science*...” (quoted right from the Abstract). But besides its style, this manuscript is well documented and seems to be methodologically sound. It formulates and substantiates a very innovative hypothetical mechanism of vasculature morphogenesis *in situ*, including enhanced glycolysis in growing tumor tissue under aerobic conditions, seemingly due to cellular heterogeneity of tumors. The strength of this study lies in the use of *in situ* methods, involving, after Frost and Borgstrom (2003), the implantation of cultured breast tumor spheroids into the healthy mouse skin fold chamber, incubation for 1 or 3 weeks *in vivo*, followed by dissecting the tissue and processing it for electron microscopy. The electron microscopy was supported by immunocytochemistry, using antibodies to identify specific molecules *in situ*. 

Unfortunately, due to methodological constraints, the redox potential in the tissue microenvironment and the rate of respiration/energy production were only indirectly inferred from the structural changes in the mitochondria, but appropriately interpreted as indicative of their temporary functional disability which correlated with mitosis. On the other hand, to my knowledge, currently there is no ethical method for direct measurement of microenvironmental tissue oxygenation and/or ATP/ADP ratio in non-anesthetized animals *in vivo*. Thus, verification of the postulated hypothetical mechanism may need to wait until such a method will be developed. 

While the title seems to be appropriate for the content of the article, the abstract deserves a revision to more clearly describe the biological system investigated, the methods used, the results obtained and the hypothetical mechanism postulated. Even though the article is relatively well constructed, its clarity could be further improved by separating the results observed by the authors from interpretations based on reports by other researchers. In any event, the interpretations and the postulated mechanism are sensible and well documented.

I have read this submission. I believe that I have an appropriate level of expertise to confirm that it is of an acceptable scientific standard.

# Peer review: Referee response for version 1

**Published:** 2013-01-28

**Authors:** Ralph DeBerardinis

**Affiliations:** 1 The University of Texas Southwestern Medical Center, Dallas, Texas, USA

The authors provide an interesting and detailed description of the ultrastructure of breast tumor spheroids ectopically implanted into immunocompromised mice. They describe numerous changes in mitochondrial and peroxisomal structure during the development of tumors in this model. Although the structural descriptions are interesting, there are limitations to the conclusions about metabolism that may be drawn from this type of analysis.  For example, changes in mitochondrial appearance do not necessarily force an increase in the glycolytic rate, as suggested. Other conclusions drawn from the microscopy studies, including the presence of hypoxia at the level of single cells, and the origin of individual cells within weeks-old tumor structures, are not well validated.

I have read this submission. I believe that I have an appropriate level of expertise to confirm that it is of an acceptable scientific standard, however I have significant reservations, as outlined above.

# Response

**Published:** 2013-04-10

**Authors:** Halina Witkiewicz

**Affiliations:** PRISM, USA

A variety of metabolic pathways can be activated in response to hypoxia. However, glycolysis is the most likely one in cells undergoing erythrogenesis because the erythrosomes use it. The main point though is that the changes in mitochondrial structure do indicate impairment of their function – the oxidative phosphorylation, and therefore imply alternative pathways. The correlation between structural integrity of mitochondria and their functioning has been documented in the literature. One more example is PM:10717001. The ultrastructural analysis *in situ* is the best way to validate hypoxia at the level of single cells. 

 The origin of individual cells was validated in the accompanying articles by immunocytochemical detection of tumor and graft cells harboring GFP coding sequences under histone H2B or ubiquitin promoter, respectively. Once integrated into the host genome the exogenous sequence was inherited by all progeny cells and expressed under control of the particular promoter. Therefore the time of tumor incubation in the chambers was irrelevant for the detection of the label. In this article the distinction between the two possible sources of HSCs was based on the location where the early stages of vasculature formation were seen - within or outside the encapsulated tumor nodule. While our conclusions based on those interesting images appeared correct to us, the readers may be inspired by the same images to formulate their own conclusions, and perhaps, build on them to develop and further test some alternative hypotheses. For the considerations on the significance of Warburg effect most important was the side by side co-existence of cells with variable metabolism, not their origin.
